# Hyperlipidemia and apolipoprotein E are associated with intraocular pressure of thyroid-associated ophthalmopathy in a Chinese population: a cross-sectional study

**DOI:** 10.3389/fendo.2024.1484343

**Published:** 2024-11-28

**Authors:** Yu Chen, Xin Qi, Jingya Wang, Huayang Xu, Yushi Sun, Ling Wang, Xingchen Zhou, Mingqian He, Jiarui Zhao, Jinbo Zhang, Hairong He, Hui Guo, Bingyin Shi, Yue Wang, Meng Zhang

**Affiliations:** ^1^ Department of Endocrinology, The First Affiliated Hospital of Xi’an Jiaotong University, Xi’an, Shaanxi, China; ^2^ Precision Medicine Center, The First Affiliated Hospital of Xi’an Jiaotong University, Xi’an, Shaanxi, China; ^3^ Department of Gastroenterology, Xi’an Children’s Hospital, Xi’an, Shaanxi, China; ^4^ Clinical Research Center, The First Affiliated Hospital of Xi’an Jiaotong University, Xi’an, Shaanxi, China

**Keywords:** thyroid-associated ophthalmopathy, hyperlipidemia, intraocular pressure, apolipoprotein E, Graves’ ophthalmopathy

## Abstract

**Objective:**

This study aimed to explore the clinical characteristics of thyroid-associated ophthalmopathy (TAO) with hyperlipidemia and to identify the key lipid indicator.

**Methods:**

Patients with TAO were recruited to this study and divided into two groups based on the presence of hyperlipidemia. TAO patients with hyperlipidemia were further classified based on the type of hyperlipidemia. Basic and clinical information of the patients were collected, and comparisons between groups were carried out. Correlation analyses, regression analyses, and stratified analysis were performed to assess the correlations and relationship of the serum lipids with the ophthalmic indicators.

**Results:**

A total of 273 patients with TAO were recruited, including 158 patients with hyperlipidemia and 115 patients without hyperlipidemia. Patients with hyperlipidemia, especially those with mixed hyperlipidemia, exhibited high intraocular pressure (IOP). Spearman’s correlation analysis and partial correlation analysis showed that apolipoprotein E (ApoE) was positively related to IOP levels after controlling for confounding factors, including age, gender, BMI, smoking history, triiodothyronine (T3), and thyrotropin (thyroid-stimulating hormone, TSH). Moreover, multiple linear regression obtained a regression equation including ApoE, gender, age, and BMI and showed that elevated ApoE levels were associated with elevated IOP [*β* = 0.072, 95% confidence interval (CI) = 0.037–0.155, *p* = 0.030]. Stratified analysis highlighted the impact of ApoE on IOP in younger patients (≤48 years), female patients, patients with normal BMI (<24 kg/m^2^), and patients with a shorter course of ophthalmopathy (≤6 months).

**Conclusion:**

Overall, higher IOP levels were observed in patients with hyperlipidemia, especially those with mixed hyperlipidemia. Notably, a higher ApoE was identified as an independent risk factor for higher IOP. This finding confirmed the close relationship between TAO and lipid metabolism and provides a new insight into the pathogenesis research and the long-term management of TAO.

## 1 Introduction

Thyroid-associated ophthalmopathy (TAO), also called thyroid eye disease (TED), is one of the common organ-specific autoimmune diseases and is closely related to Graves’ hyperthyroidism (GH) (1, 2). Chronic autoimmune inflammation of the orbital and periorbital tissues is the main pathological characteristic of TAO, causing adipogenesis, extraocular muscle (EOM) inflammation, fibrosis, and irreversible tissue remodeling (3, 4). TAO can manifest itself through various clinical presentations, including proptosis, diplopia, and visual impairment (5–8). In addition, 3.1%–24% of patients with TAO manifested ocular hypertension, which could lead to the development of glaucomatous damage (2). Current treatments, including glucocorticoids (GCs), radiotherapy, and immunosuppressants, struggle to achieve satisfactory efficacy and carry a risk of severe side effects, leading to the continuous exploration of new mechanisms and treatments for TAO (9).

The relationship between lipid metabolism and TAO has been gradually recognized in recent years. In 2018, a cross-sectional study and a prospective study both confirmed the association between total cholesterol (TC) and low-density lipoprotein cholesterol (LDL-c) and the occurrence of TAO; however, their relationship with clinical activity remains inconsistent (10, 11). Triglycerides (TGs) have been reported to affect the response of patients with TAO to GCs and to predict the effectiveness by combining orbital MRI parameters (12, 13). Multiple clinical studies have confirmed that lipid-lowering drugs, statins, could decrease the incidence of TAO in patients with GH, indicating the important role of lipid metabolism in the pathogenesis of TAO (9, 14–16). Mechanistic studies have shown that immune cells (such as T helper 17 cells) and the gut microbiota (such as *Klebsiella pneumoniae*) may be involved (17, 18). However, the clinical characteristics of TAO patients with hyperlipidemia have not been systematically examined and deserve further exploration and research.

To better explore the clinical characteristics of TAO patients with hyperlipidemia, 273 patients with TAO were enrolled in the study and grouped based on the presence and the subtypes of hyperlipidemia. The basic and clinical indicators of TAO were compared between groups. Correlation and linear regression analyses were performed to explore the associations between serum lipids and ophthalmic indicators. This research aimed to provide new insights for further studies on the pathogenesis and clinical management for TAO.

## 2 Materials and methods

### 2.1 Participants

Patients with TAO admitted to the Department of Endocrinology from 2010 to 2019 were invited for participation. All of the patients were over 18 years old and were diagnosed with TAO by professional clinician based on clinical manifestations and the orbital CT results. The exclusion criteria were: a) concurrent extra-thyroid autoimmune diseases; b) concurrent severe heart failure (New York Heart Association classes II–IV), severe liver insufficiency (Child–Pugh classes B/C), and kidney insufficiency (eGFR ≤59 mL/min) (19, 20); c) concurrent malignant tumors; d) concurrent severe ocular diseases such as inflammatory pseudotumors; e) treatment with orbital radiotherapy, decompression surgery, oral GCs, or other immunosuppressive therapy within the previous 3 months; and f) incomplete data on ophthalmic assessments and/or serum lipid. Finally, 273 patients were enrolled (**Supplementary Figure 1**).

### 2.2 Data collection

All of the participants enrolled in the study signed informed consent after fully understanding the study protocol. An electronic medical record system was used to collect the basic information, anthropometric measurements, medication history, ophthalmic assessments, and the laboratory tests of patients. Basic information included age, gender, and smoking status. Disease history included thyroid dysfunction, diabetes, hypertension, and the duration of the eye disease. Anthropometric measurements included body mass index (BMI), systolic blood pressure (SBP), and diastolic blood pressure (DBP). Medication history included the use of anti-thyroid drugs (ATDs), radioactive iodine therapy (RAI), and anti-lipid medications. Ophthalmic assessments included the presence of diplopia, the clinical activity score (CAS) for disease activity, the NOSPECS score (no TAO sign, only eyelid sign, soft tissue involvement, proptosis, extraocular motility restriction, corneal involvement, sight loss) for disease severity, visual acuity (VA), intraocular pressure (IOP), and exophthalmometry (21). In particular, the E Standard Logarithmic Visual Acuity Chart (GB 11533-1989) was used to measure VA at a distance of 5 m (22). The corresponding relationship between the E chart and the Snellen chart is as follows: 0.1, 0.2, 0.4, 0.8, and 1 in the E chart are equivalent to 20/200, 20/100, 20/50, 20/25, and 20/20 in the Snellen chart, respectively. Considering the possibility of a unilateral onset of TAO, for patients with bilateral data for vision, IOP, and exophthalmometry, the more severe values were used. Laboratory test results included thyroid function indicators and lipid profiles, all of which were measured using fasting venous blood samples collected in the morning after an 8-h fast.

### 2.3 Group definitions

According to the 2016 “Chinese Guidelines for the Management of Dyslipidemia in Adults,” hyperlipidemia is diagnosed and categorized into four types (23): hypertriglyceridemia (TG ≥ 1.7 mmol/L and TC < 5.2 mmol/L), hypercholesterolemia (TG < 1.7 mmol/L and TC ≥ 5.2mmol/L), mixed hyperlipidemia (TG ≥ 1.7 mmol/L and TC ≥ 5.2 mmol/L), and low high-density lipoprotein cholesterol (HDL-c) (TG < 1.7 mmol/L, TC < 5.2mmol/L, and HDL-c < 1.0 mmol/L).

### 2.4 Statistical analysis

Statistical analysis was performed using SPSS 25.0 software. Normally distributed data were expressed as the mean ± standard deviation (SD), with Student’s *t*-test used for group comparisons. Abnormally distributed data were reported as median and interquartile range (IQR), with the Mann–Whitney *U* test used for group comparisons. Categorical data were expressed as proportions, and the chi-squared test was used for group comparisons. Spearman’s correlation analysis and partial correlation analysis were performed to assess the correlations of the serum lipids with the indicators of ophthalmic assessments. Linear regression was used to assess the relationship between the blood lipid indices and specific ophthalmic indicators in patients with TAO. Unless otherwise noted, a *p* < 0.05 was considered statistically significant.

## 3 Results

### 3.1 Clinical characteristics of the study participants

#### 3.1.1 Clinical characteristics of the TAO patients with hyperlipidemia

A total of 273 patients with TAO were enrolled in the study, comprising 158 patients with hyperlipidemia (57.9%) and 115 patients without hyperlipidemia (42.1%). The basic clinical information of the patients in the two groups is shown in **Table 1**.

**Table 1:** Clinical characteristics of thyroid-associated ophthalmopathy (TAO) patients with or without hyperlipidemia.

	Total (*N* = 273)	Without hyperlipidemia (*n* = 115)	With hyperlipidemia (*n* = 158)	*p*-value
Men, *n* (%)	155 (56.8)	50 (43.5)	105 (66.5)	**<0.001**
Age (years)	48.17 ± 11.05	46.13 ± 12.34	49.65 ± 9.77	**0.012**
Smoking status, *n* (%)				**<0.001**
Non-smokers	172 (63.0)	86 (74.8)	86 (54.4)	
Ex-smokers	21 (7.7)	3 (2.6)	18 (11.4)	
Current smokers	80 (29.3)	26 (22.6)	54 (34.2)	
BMI (kg/m^2^)	22.9 (21.0–25.2)	21.5 (19.9–24.5)	23.3 (21.6–26.0)	**<0.001**
SBP (mmHg)	117 (110–130)	113 (107–124)	120 (110–138)	**0.004**
DBP (mmHg)	73 (68–80)	70 (64–80)	77 (70–83)	**0.005**
History of hypertension, *n* (%)^a^	45 (18.3)	14 (13.9)	31 (21.4)	0.129
History of diabetes, *n* (%)^a^	22 (9.0)	5 (5)	17 (11.7)	0.058
Anti-lipid drugs, *n* (%)^a^	11 (4.5)	1 (1.0)	10 (7.6)	**0.009**
Thyroid dysfunction, *n* (%)				0.825
Euthyroid	10 (3.7)	4 (3.5)	6 (3.8)	
Graves’ disease	174 (63.7)	76 (66.1)	98 (62)	
Hashimoto’s thyroiditis	42 (15.4)	15 (13)	27 (17.1)	
Other^b^	47 (17.2)	20 (17.4)	27 (17.1)	
Duration of Graves’ disease (months)	10 (6–18)	12 (6–24)	8 (5–18)	0.083
ATDs, *n* (%)	191 (70)	81 (71.3)	110 (69.6)	0.749
Imidazoles, *n* (%)	172 (63.0)	74 (64.3)	98 (62.0)	
Thioureas, *n* (%)	19 (7.0)	7 (6.1)	12 (7.6)	
RAI, *n* (%)	18 (6.6)	7 (6.1)	11 (7)	0.774
Positive TPOAb, *n* (%)	169 (61.9)	75 (65.2)	94 (49.5)	0.335
Positive TGAb, *n* (%)	73 (26.7)	27 (23.5)	46 (29.1)	0.297
Positive TMAb, *n* (%)	69 (25.3)	25 (21.7)	44 (27.8)	0.249
T4 (μg/dL)	8.22 (6.44–10.8)	8.25 (6.19–10.9)	8.15 (6.5–10.7)	0.667
T3 (ng/mL)	1.47 (1.13–1.84)	1.51 (1.13–1.85)	1.43 (1.13–1.82)	0.53
FT4 (pmol/L)	15.9 (13.4–18.6)	16 (14–19.8)	15.45 (13–18.23)	0.185
FT3 (pmol/L)	5.47 (4.64–6.81)	5.5 (4.72–6.63)	5.39 (4.6–6.93)	0.572
TSH (μIU/mL)	0.18 (0.07–2.1)	0.127 (0.07–1.57)	0.24 (0.07–2.22)	0.529
Lipid profile				
TC (mmol/L)	4.18 ± 1.002	3.93 ± 0.634	4.36 ± 1.17	**<0.001**
TG (mmol/L)	1.27 (0.88–1.8)	0.91 (0.68–1.22)	1.67 (1.18–2.17)	**<0.001**
HDL-C (mmol/L)	1.1 (0.92–1.31)	1.21 (1.10–1.36)	0.95 (0.83–1.20)	**<0.001**
LDL-C (mmol/L)	2.38 (1.93–2.95)	2.12 (1.81–2.62)	2.7 (1.99–3.26)	**<0.001**
Apo A (g/L)	1.20 (1.07–1.34)	1.27 (1.18–1.37)	1.11 (0.98–1.29)	**<0.001**
Apo B (g/L)	0.78 (0.62–0.91)	0.7 (0.59–0.83)	0.83 (0.66–0.97)	**<0.001**
Apo E (mg/L)	33.3 (28.9–41.8)	31.9 (28.3–38.1)	35.8 (30.2–43.8)	**0.011**
Lp(a) (mg/L)	110.6 (53.8,193.6)	120.6 (61.6–213.4)	98.3 (47.4–190.3)	0.088
Clinical features				
Duration of oculopathy (months)	6 (3–12)	6 (3–12)	6 (4–12)	0.752
CAS	3 (3–4)	3 (2–4)	3 (3–4)	0.202
NOSPECS	4 (4–4)	4 (4–4)	4 (4–4)	0.228
Diplopia (%)	190 (69.6)	70 (60.9)	120 (75.8)	**0.008**
VA	0.6 (0.5–1.0)	0.8 (0.5–1.0)	0.6 (0.4–1.0)	0.433
Exophthalmometry (mm)	18 (16–20)	19 (17–20)	18 (16–21)	0.722
IOP (mmHg)	19 (16–22)	18 (15.9–21.5)	19 (17–22.8)	**0.045**

BMI, body mass index; SBP, systolic blood pressure; DBP, diastolic blood pressure; ATDs, anti-thyroid drugs; RAI, radioactive iodine therapy; TPOAb, thyroid peroxidase antibodies; TGAb, thyroglobulin antibodies; TMAb, thyroid microsomal antibodies; T4, thyroxine; T3, triiodothyronine; FT4, free thyroxine; FT3, free triiodothyronine; TSH, thyrotropin; TG, triglyceride; TC, total cholesterol; ApoA, apolipoprotein A; ApoB, apolipoprotein B; ApoE, apolipoprotein E; Lp(a), lipoprotein(a); HDL-c, high-density lipoprotein cholesterol; LDL-c, low-density lipoprotein cholesterol; CAS, clinical activity score; VA, visual acuity; IOP, intraocular pressure.

a Missing values in less than 10% of patients.

b Unclear or other thyroid dysfunction.

Bold values: significant differences (P value of less than 0.05).

Compared with the group of TAO patients without hyperlipidemia, the group with hyperlipidemia had more male patients, more patients with a smoking history, were of older age, had higher BMI and high blood pressure, and had more patients treated with anti-lipid drugs, with statistical significance (*p* < 0.05). There were no differences between the two groups in terms of history of diabetes, hypertension and thyroid dysfunction, duration of Graves’ disease, use of ATDs and RAI, and thyroglobulin antibodies (TGAb), thyroid microsomal antibodies (TMAb), and thyroid peroxidase antibodies (TPOAb), as well as all of the thyroid function indicators (*p* > 0.05). TAO patients with hyperlipidemia exhibited higher TC, TG, LDL-c, apolipoprotein A (ApoA), apolipoprotein B (ApoB), and apolipoprotein E (ApoE) levels, as well as lower HDL-c levels, compared with TAO patients without hyperlipidemia (*p* < 0.05).

In terms of ophthalmic indicators, the IOP levels of TAO patients with hyperlipidemia were significantly higher than those of TAO patients without hyperlipidemia [19 (17–22.8) vs. 18 (15.9–21.5) mmHg, *p* = 0.045], as well as the incidence of diplopia (75.8% vs. 60.9%, *p* = 0.008). There were no significant differences between the two groups in terms of duration of ophthalmopathy, CAS, NOSPECS, vision, and exophthalmometry.

#### 3.1.2 Clinical characteristics of TAO patients with different phenotypes of hyperlipidemia

Subsequently, the clinical characteristics of TAO in patients with different types of hyperlipidemia were explored. The clinical information is shown in **Table 2**. Different phenotypes of hyperlipidemia exhibited different lipid profiles. In terms of basic indices, compared with TAO patients without hyperlipidemia, those with different types of hyperlipidemia have different characteristics in terms of age, gender, BMI, smoking status, blood pressure, history of hypertension, and use of anti-lipid drugs (*p* < 0.05). TAO patients with hypertriglyceridemia had lower free thyroxine (FT4) levels (*p* = 0.026), while TAO patients with mixed hyperlipidemia had lower free triiodothyronine (FT3) levels (*p* = 0.005) and higher thyrotropin (thyroid-stimulating hormone, TSH) levels (*p* = 0.037).

**Table 2:** Clinical characteristics of thyroid-associated ophthalmopathy (TAO) patients with different types of hyperlipidemia.

Group	Hypercholesterolemia (*n* = 22)	Hypertriglyceridemia (*n* = 59)	Mixed hyperlipidemia (*n* = 19)	Low high-density lipoproteinemia (*n* = 58)
Men, *n* (%)	14 (63.6)	39 (66.10)***	6 (31.6)	46 (79.3)***
Age (years)	47.36 ± 9.34	50.12 ± 9.60*	50.74 ± 6.92*	49.69 ± 10.92
Smoking status, *n* (%)		<0.001***		0.008**
Non-smokers	11 (50.0)	30 (50.8)	15 (78.9)	30 (51.7)
Ex-smokers	2 (9.1)	10 (16.9)	1 (5.3)	5 (8.6)
Current smokers	9 (40.9)	19 (32.2)	3 (15.8)	23 (39.7)
BMI (kg/m^2^)	23.9 (22.0–28.1)	23.4 (21.7–27.7)***	23.1 (21.2–25.0)**	23.9 (22.0–28.1)**
SDP (mmHg)	134 (117–146)**	116 (110–130)	120 (110–143)	120 (111–140)*
DBP (mmHg)	81 (76–91)**	72 (69–80)	79 (70–90)*	78 (69–85)
History of hypertension, *n* (%)^a^	4 (21.1)	15 (28.8)*	4 (21.1)	8 (14.5)
History of diabetes, *n* (%)^a^	2 (10.5)	6 (11.5)	2 (10.5)	7 (12.7)
Anti-lipid drugs, *n* (%)^a^	0 (0.0)	6 (11.5)**	4 (21.1)**	1 (1.8)
Thyroid dysfunction, *n* (%)				
Euthyroid	2 (9.1)	4 (6.7)	0 (0.0)	1 (1.7)
Graves’ disease	11 (50)	32 (53.3)	13 (68.4)	42 (72.4)
Hashimoto’s thyroiditis	4 (18.2)	8 (13.3)	5 (26.3)	10 (17.2)
Other^b^	5 (22.7)	16 (26.7)	1 (5.3)	5 (8.6)
Duration of Graves’ disease (months)	6 (4–12)*	12 (6–36)	5 (4–13)	10 (6–19)
ATDs, *n* (%)	18 (81.8)	40 (67.8)	13 (68.4)	42 (72.4)
Imidazoles, *n* (%)	16 (72.7)	34 (57.6)	10 (52.6)	38 (65.5)
Thioureas, *n* (%)	2 (9.1)	4 (6.8)	3 (15.8)	3 (5.2)
RAI, *n* (%)	3 (13.6)	3 (5.1)	3 (15.8)	2 (3.4)
Positive TPOAb, *n* (%)	14 (63.6)	25 (42.4)**	14 (73.7)	41 (70.7)
Positive TGAb, *n* (%)	4 (18.2)	15 (25.4)	4 (21.1)	22 (37.9)*
Positive TMAb, *n* (%)	4 (18.2)	15 (25.4)	4 (21.1)	21 (36.2)*
T4 (μg/dL)	5.97 (4.73–6.99)	5.42 (4.56–6.61)	4.74 (3.71–5.58)	5.97 (4.73–6.99)
T3 (ng/mL)	1.39 (1.07–1.69)	1.34 (1.12–1.61)	1.40 (1.01–1.54)	1.67 (1.25–2.37)
FT4 (pmol/L)	15.25 (13–17.23)	14.95 (12.48–16.7)*	14.95 (13.23–17.48)	16.85 (12.98–25.7)
FT3 (pmol/L)	5.97 (4.73–6.99)	5.42 (4.56–6.61)	4.74 (3.71–5.58)**	5.97 (4.73–6.99)
TSH (μIU/mL)	0.88 (0.07–3.64)	0.37 (0.07–2.14)	1.18 (0.78–6.77)*	0.07 (0.07–1.05)
Lipid profile				
TC (mmol/L)	5.62 (5.44–5.95)***	4.21 (3.66–4.56)	5.82 (5.45–6.24)***	3.61 (2.98–4.20)**
HDL (mmol/L)	1.41 (1.27–1.71)***	0.92 (0.79–1.07)***	1.27 (1.08–1.44)	0.90 (0.81–0.95)***
TG (mmol/L)	1.18 (0.96–1.55)***	2.17 (1.91–2.81)***	2.32 (1.83–2.99)***	1.17 (0.90–1.39)***
LDL (mmol/L)	3.89 (3.29–4.16)***	2.43 (2.05–2.84)**	3.74 (3.49–4.48)***	1.97 (1.63–2.50)
ApoA (g/L)	1.35 (1.33–1.56)**	1.14 (0.99–1.26)***	1.39 (1.27–1.57)**	1.01 (0.94–1.10)***
ApoB (g/L)	1.05 (0.95–1.15)***	0.83 (0.71–0.90)***	1.15 (1.07–1.24)***	0.67 (0.51–0.83)
ApoE (mg/L)	41.1 (33.9–50.3)**	39.3 (32.3–45.1)***	49.5 (41.2–81.2)***	29.3 (23.8–34.2)**
Lp(a) (mg/L)	119.8 (57.0–244.6)	79.2 (45.6–145.7)*	216.3 (136.6–438.1)*	98.6 (39.0–187.5)
Clinical features				
Duration of oculopathy (months)	5 (2–8.8)	6 (4–12)	6 (3–6)	6(4–12)
CAS	3 (3–4)	3 (3–4)	3 (3–4)*	3 (3–4)
NOSPECS	4 (4–4)	4 (4–4)	4 (4–4)	4 (4–4.25)
Diplopia, *n* (%)	16 (72.7)	47 (79.7)	13 (68.4)	44 (75.9)
VA	0.7 (0.3–1.0)	0.7 (0.5–1.0)	0.5 (0.475–0.8)	0.6 (0.4–0.85)
Exophthalmometry (mm)	20 (17.5–21.5)	18 (16–21)	19 (15.5–22)	17 (16–20)
IOP (mmHg)	20.5 (18–22)	19.5 (17–23.4)	21 (19–23)*	18 (15.8–21.6)

BMI, body mass index; SBP, systolic blood pressure; DBP, diastolic blood pressure; ATDs, anti-thyroid drugs; RAI, radioactive iodine therapy; TPOAb, thyroid peroxidase antibodies; TGAb, thyroglobulin antibodies; TMAb, thyroid microsomal antibodies; T4, thyroxine; T3, triiodothyronine; FT4, free thyroxine; FT3, free triiodothyronine; TSH, thyrotropin; TG, triglyceride; TC, total cholesterol; ApoA, apolipoprotein A; ApoB, apolipoprotein B; ApoE, apolipoprotein E; Lp(a), lipoprotein(a); HDL-c, high-density lipoprotein cholesterol; LDL-c, low-density lipoprotein cholesterol; CAS, clinical activity score; VA, visual acuity; IOP, intraocular pressure.

*p < 0.05, **p < 0.01, ***p < 0.001 (compared with TAO patients without hyperlipidemia).

a Missing values in less than 10% of patients.

b Unclear or other thyroid dysfunction.

Bold values: significant differences (P value of less than 0.05).

In terms of the indicators of ophthalmic assessments, the IOP of patients with hyperlipidemia was higher than that of non-hyperlipidemia patients (*p* = 0.045), as well as CAS (*p =* 0.024).

### 3.2 Correlation analysis of the serum lipid indices and ophthalmic indicators of TAO

#### 3.2.1 Spearman’s correlation analysis of the serum lipid indices and ophthalmic indicators of TAO

**Figure 1:**
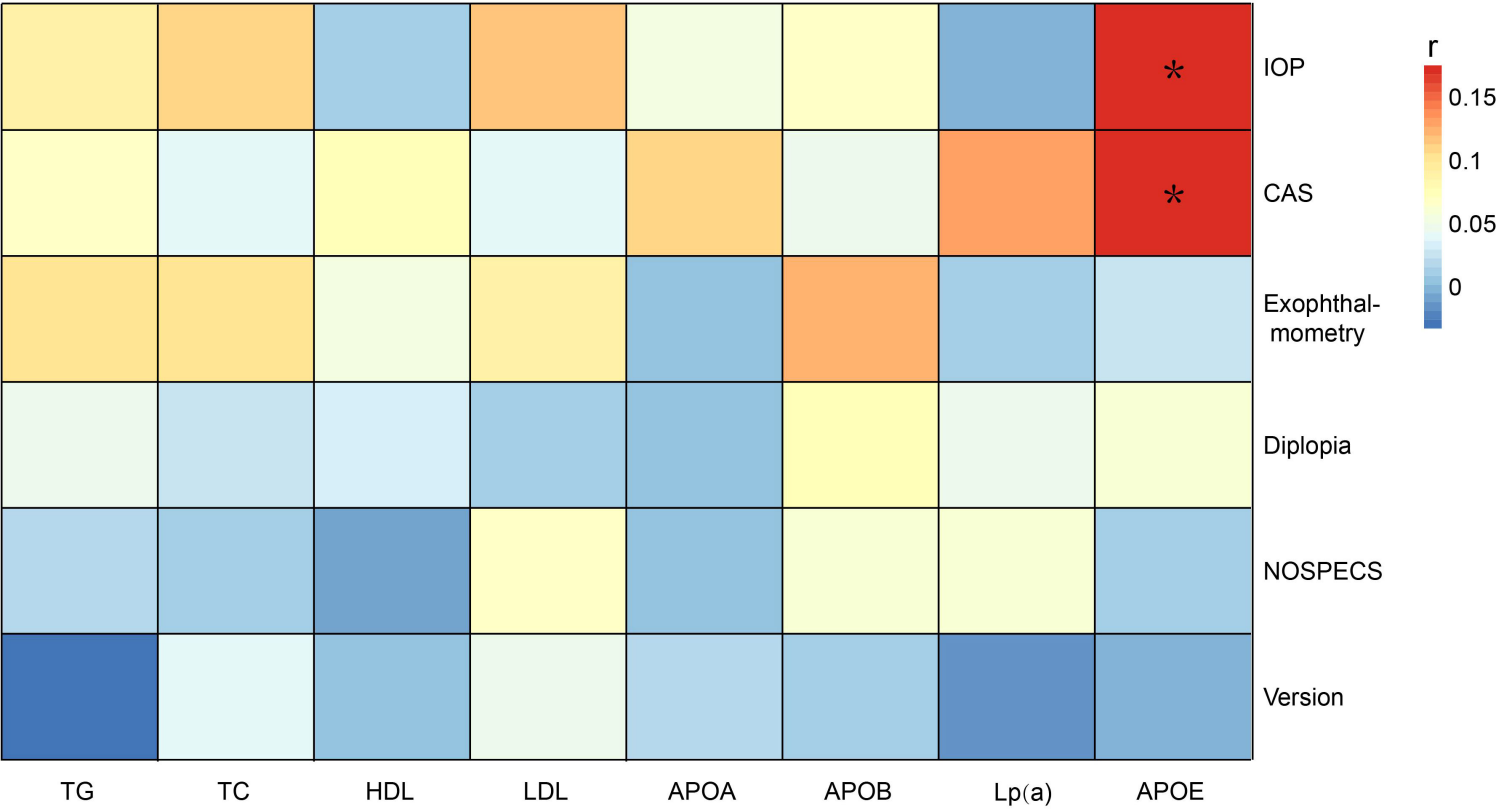
Correlation analysis of the serum lipid indices and ophthalmic indicators of thyroid-associated ophthalmopathy (TAO) adjusted for age and gender. *p < 0.05. TG, triglyceride; TC, total cholesterol; ApoA, apolipoprotein A; ApoB, apolipoprotein B; ApoE, apolipoprotein E; Lp(a), lipoprotein(a); HDL-c, high-density lipoprotein cholesterol; LDL-c, low-density lipoprotein cholesterol; CAS, clinical activity score; IOP, intraocular pressure.

Spearman’s correlation analysis between the serum lipid indices and ophthalmic indicators of TAO was performed. The age and gender were controlled considering the significant differences in basic data between the two groups, as shown in **Table 1** (**Figure 1**). The results showed that, after controlling for age and gender, ApoE exhibited a positive correlation with both the CAS and IOP level [*r* = 0.175 (*p* = 0.018) and *r* = 0.174 (*p* = 0.018), respectively]. This indicated that ApoE was positively correlated with IOP level and CAS.

#### 3.2.2 Partial correlation analysis of ApoE and the ophthalmic indicators

To control for confounding factors, the relationship between the ApoE and IOP levels and CAS was further explored using partial correlation analysis. Three models were established: model 1 controlled for age, gender, BMI, and smoking status; model 2 controlled for age, gender, BMI, smoking status, history of hypertension, history of diabetes, and anti-lipid drugs; and model 3 controlled for age, gender, BMI, smoking status, history of hypertension, history of diabetes, anti-lipid drugs, TPOAb, TGAb, TMAb, FT3, and TSH. The results showed that, in model 3, which controlled for multiple factors, ApoE still exhibited a significant positive correlation with IOP (*r* = 0.210, *p* = 0.022) (**Table 3**).

**Table 3:** Partial correlation analysis of apolipoprotein E (ApoE) and the ophthalmic indicators.

	Model 1		Model 2		Model 3	
	*r*	*p*	*r*	*p*	*r*	*p*
IOP	**0.174**	**0.036**	**0.193**	**0.024**	**0.210**	**0.020**
CAS	0.037	0.655	0.001	0.993	0.022	0.806

Model 1: adjusted for age, body mass index (BMI), and smoking status; model 2: adjusted for age, gender, BMI, smoking status, history of hypertension, history of diabetes, and anti-lipid drugs; model 3: adjusted for age, gender, BMI, smoking status, history of hypertension, history of diabetes, anti-lipid drugs, thyroid peroxidase antibodies (TPOAb), thyroglobulin antibodies (TGAb), thyroid microsomal antibodies (TMAb), free triiodothyronine (FT3), and thyrotropin (TSH).

CAS, clinical activity score; IOP, intraocular pressure.

Bold values: significant differences (P value of less than 0.05).

### 3.3 Regression analysis of IOP

#### 3.3.1 Linear regression analysis of IOP

The findings above demonstrated that the lipid indices, particularly ApoE, are correlated with IOP. Therefore, a linear regression analysis was performed for IOP. Firstly, taking the IOP level as the dependent variable and adjusting for age and gender, we performed regression analysis on BMI, smoking history, SBP, DBP, thyroid function indicators, and lipid indices. The results indicated that BMI, FT4, TC, TG, LDL-c, and ApoE are potential risk or protective factors for IOP (*p* < 0.1) (**Supplementary Table 1**).

**Figure 2:**
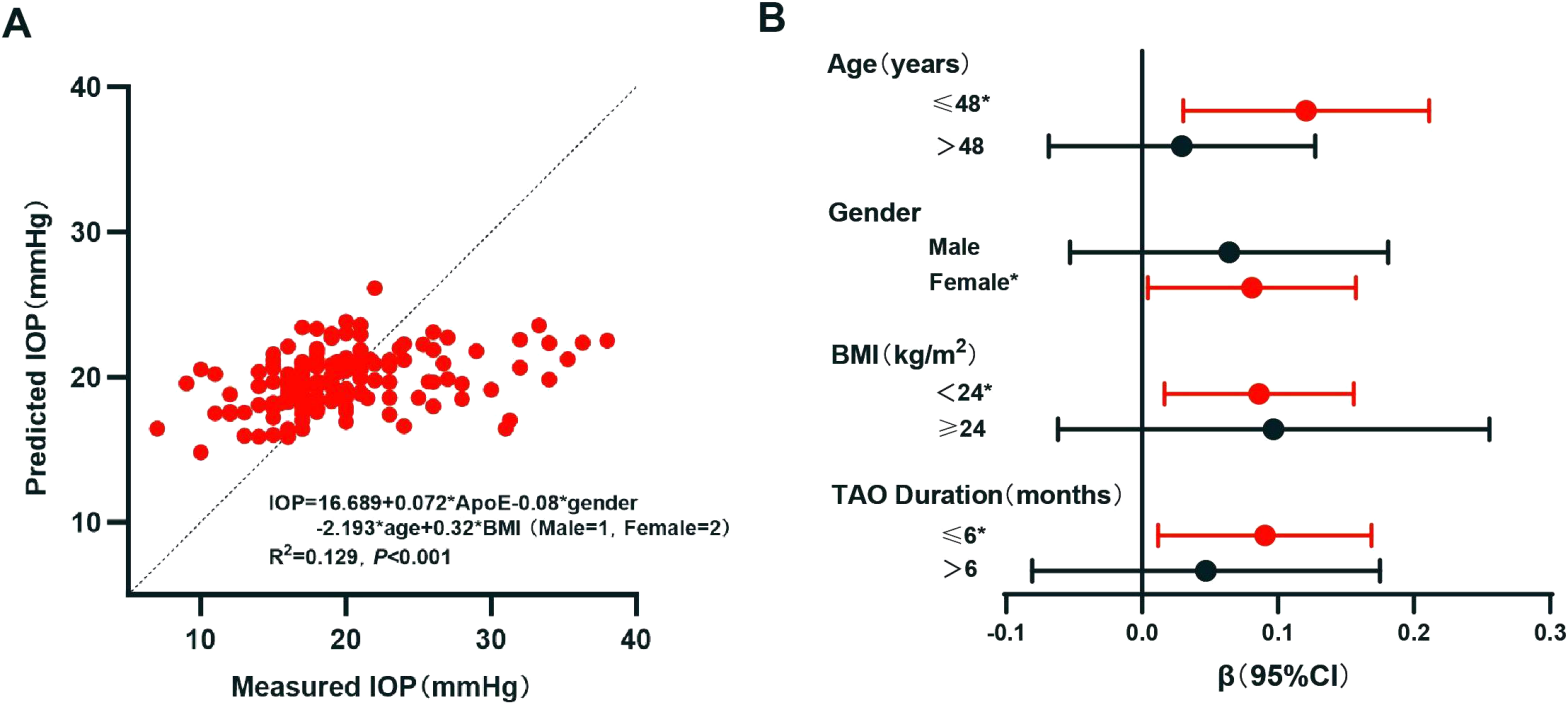
Linear regression analysis of intraocular pressure (IOP). **(A)** Multivariate linear regression analysis of IOP. **(B)** Linear regression of IOP with apolipoprotein E (ApoE) as a variable in different groups: different age groups were adjusted for gender and body mass index (BMI); different gender groups were adjusted for age and BMI; different BMI groups were adjusted for age and gender; and different thyroid-associated ophthalmopathy (TAO) durations were adjusted for age, gender, and BMI. *p < 0.05. *CI*, confidence interval.

Subsequently, the IOP level was taken as the dependent variable and the above indicators (i.e., age, gender, BMI, FT4, TC, TG, LDL-c, and ApoE) included as independent variables in a multivariate linear regression analysis (backward method). The results obtained a regression equation including ApoE, gender, age, and BMI (**Table 4**; **Figure 2A**). The *p* and *β* (95% CI) values of ApoE on IOP were 0.03 and 0.72 (0.007–0.137), respectively, indicating that a higher ApoE level is the independent risk factor for a higher IOP level: for each 1 mg/L increase in ApoE, the IOP increases by 0.072 mmHg.

**Table 4:** Multivariate linear analysis of intraocular pressure (IOP).

Variate	*β*	95% CI		*p*
		Lower	Upper	
Gender	−2.193	−3.975	−0.410	0.016
Age	−0.080	−0.156	−0.002	0.043
BMI	0.32	0.047	0.593	0.022
ApoE	0.72	0.007	0.137	0.030

BMI, body mass index; ApoE, apolipoprotein E; CI, confidence interval.

#### 3.3.2 Stratified analysis by linear regression

As gender, age, and BMI were independent factors for IOP, a stratified analysis was conducted and linear regression was used to verify the stability of the effect of ApoE on IOP. As the mean age of all patients was 48.17 years, 48 years was used as the cutoff for age. With BMI ≥ 24 kg/m^2^ defined as overweight/obesity in the Chinese population, 24 kg/m^2^ was used as the cutoff for BMI (24). Duration has been reported to be involved in the relationship of lipid indicators with the onset and activity of TAO (10). Thus, stratified analysis on the duration of TAO was performed, with the median value (6 months) being used as the cutoff. The results revealed that ApoE was an independent risk factor for IOP in particular subgroups (**Figure 2B**): age ≤ 48 years, with *p* and *β* (95% CI) values of 0.01 and 0.121 (0.030–0.211), respectively; women, with *p* and *β* (95% CI) values of 0.039 and 0.081 (0.004–0.157), respectively; BMI <24 kg/m^2^, with *p* and *β* (95% CI) values of 0.029 and 0.075 (0.008–0.142), respectively; and TAO duration ≤6 months, with *p* and *β* (95% CI) values of 0.025 and 0.09 (0.012–0.169), respectively.

## 4 Discussion

In this study, higher IOP levels were demonstrated in patients with hyperlipidemia, especially those with mixed hyperlipidemia. Notably, ApoE was identified as an independent risk factor for IOP, particularly prominent in younger patients, in female patients, and in patients with normal BMI. This finding further elucidates the close relationship between TAO and lipid metabolism and provides a new insight for the long-term management of patients with TAO.

In this study, higher IOP levels were found in TAO patients with hyperlipidemia. A recent study has indicated that 29% of patients with TAO developed glaucoma caused by an elevated IOP, which was significantly higher than that in non-TAO patients (25). Patients with high IOP have also been reported to have a higher risk of developing chronic strabismus, necessitating strabismus surgery and orbital decompression surgery (26). IOP levels are related to the clinical activity and severity of TAO (27, 28), and multiple cross-sectional studies have identified gender, age, smoking status, and TGAb as potential risk factors for elevated IOP levels in patients with TAO (29, 30). The results of this study indicated that IOP is indeed influenced by basic factors such as gender, age, and BMI, while factors such as smoking, hypertension, and TGAb do not significantly impact IOP. This difference may be due to the differences in the sample size and population. Most importantly, this study is the first to report the correlation between lipid metabolism and IOP in patients with TAO. Given that an elevated IOP can lead to secondary glaucoma and chronic strabismus, this finding indicates the necessity of the regular monitoring of and a standardized intervention for lipid indices in the long-term management of patients with TAO.

ApoE was identified as an independent risk factor for elevated IOP in patients with TAO through further correlation and regression analyses. Previous large-scale studies on healthy individuals, including the UK Biobank, have shown correlations between IOP and the lipid indicators, such as TC, HDL-c, LDL-c, TG, ApoA, and ApoB, and the mechanism was thought to be involved with atherosclerosis or vascular (31–35). In our study, ApoE was identified as a more significant lipid indicator. This difference could be due to the differences in the sample size and population, especially the pathogenesis of TAO. The mechanism of elevated IOP in TAO differs from that in primary glaucoma, in which immune-inflammatory factors are involved (29). In addition to the genetic background, the pathogenesis of elevated IOP includes orbital congestion and increased episcleral venous pressure caused by immune-inflammation, restriction and compression of the eyeball caused by enlarged and fibrotic EOM, and glycosaminoglycan deposition in the trabecular meshwork (29, 36–38). As a lipoprotein, ApoE participates in the biological process of cholesterol metabolism, and its genotype has been used to guide the use of statins (39). Furthermore, recent studies have revealed that ApoE can act as a ligand and that it participates in several immune processes, including monocyte differentiation, neutrophil senescence, and microglial immune metabolism (40–42). Thus, ApoE could affect the IOP of patients with TAO through multiple pathways, including cholesterol metabolism and immune regulation, which need to be further explored.

Considering the correlations of the lipid indices with both gender and BMI, as well as the correlations of IOP with both age and gender, a stratified study on relevant patients was conducted (32, 35). The results indicated that ApoE had an impact on IOP in subgroups of female patients, patients under 48 years, and those with normal BMI. Moreover, the stratified study on the different disease durations of patients with TAO showed that ApoE was correlated with IOP in patients with shorter disease duration, which is consistent with previous results (10). Mechanism studies have shown that the pathology of TAO is progressive, which varies from the early to the late stages. Clinical studies also showed that patients with shorter disease duration were more likely to respond to treatment compared with those with longer disease duration (43, 44). Thus, the results offered insights into the mechanisms of elevated IOP in TAO and more precise recommendations for the management of patients with TAO.

In addition, a higher CAS was observed in patients with mixed hyperlipidemia. A 2018 study enrolling 133 patients with TAO indicated that TAO activity correlated with TC and LDL-c when controlling for the duration of disease (10). However, another prospective study enrolling 86 patients did not confirm the results (11). In this study, which enrolled 273 patients, a higher CAS was found in TAO patients with mixed hyperlipidemia rather than with hypercholesterolemia. Previous research has shown that, compared with other lipid-lowering drugs, statins, which lower both TC and TG, have a preventive effect on TAO (9, 14, 15). This suggests that the relationship between the lipid levels and clinical activity might involve multiple lipid indicators such as TG and TC. Further research is needed to confirm and explore the underlying mechanism.

This study has several strengths. Firstly, to our knowledge, this is the first article analyzing the clinical characteristics of TAO based on the presence and the type of hyperlipidemia. Secondly, for the first time, we demonstrated a correlation between hyperlipidemia and higher IOP in TAO and identified ApoE as an independent risk factor for the IOP levels. Moreover, stratified analysis revealed that the impact of ApoE on IOP is more pronounced in subgroups of patients who are women, young, and with normal BMI. In addition, this study found that mixed hyperlipidemia, rather than simple hypercholesterolemia, is associated with higher clinical activity of patients with TAO, providing new insights into the relationship between lipid levels and CAS.

The study has some limitations. Firstly, this is a cross-sectional study in the northwest of China; therefore, the causal relationship could not be determined and the study might have limited guiding significance for other populations. Secondly, the patients in this study were all from inpatient departments and had high clinical activity and severity, which could limit the guiding significance for patients with low clinical activity and severity. Thirdly, the structural data of the eyes, including xanthelasma or corneal arcus, have not been studied, and this could be further explored.

In conclusion, it was demonstrated that TAO patients with hyperlipidemia exhibited higher IOP levels and that TAO patients with mixed hyperlipidemia also exhibited a higher clinical activity. Moreover, ApoE was identified as an independent risk factor for high IOP levels. This finding provides new insights into the pathogenesis of TAO and suggests the importance of the regular monitoring of and a standardized intervention for serum lipid in the management of patients with TAO.

## Data Availability

The raw data supporting the conclusions of this article will be made available by the authors, without undue reservation.

## Supplementary material

Supplementary Figure 1 Flowchart of the study. TAO, thyroid-associated ophthalmopathy.
