# Supplementary figures and images for: Hyperlipidemia and apolipoprotein E are associated with intraocular pressure of thyroid-associated ophthalmopathy in a Chinese population: a cross-sectional study
Source: Front Endocrinol (Lausanne). 2024 Nov 28;15:1484343. doi: 10.3389/fendo.2024.1484343 (PMC11634611; doi:10.3389/fendo.2024.1484343)

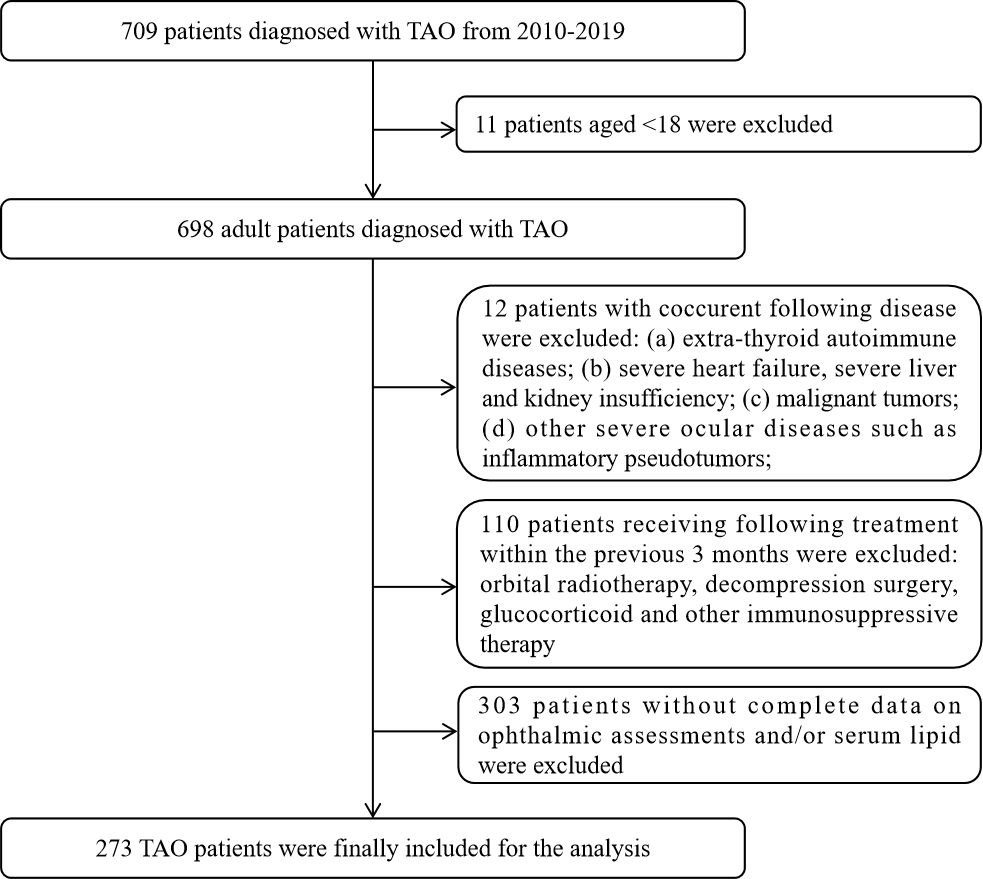

Supplement: Supplementary Figure 1 — Flowchart of the study. TAO, thyroid-associated ophthalmopathy. [file Image1.png]
