# Supplementary material for: Hyperlipidemia and apolipoprotein E are associated with intraocular pressure of thyroid-associated ophthalmopathy in a Chinese population: a cross-sectional study
Source: Front Endocrinol (Lausanne). 2024 Nov 28;15:1484343. doi: 10.3389/fendo.2024.1484343 (PMC11634611; doi:10.3389/fendo.2024.1484343)
Supplement: Supplementary file 2 [file Table1.docx]

Supplementary Material

# Supplementary TableS1

**TableS1 Univariate linear regression analysis on IOP adjusted for age and gender**

| **Variate** | **β** | **95CI** | | ***P*** | **Variate** | **β** | **95CI** | | ***P*** |
| --- | --- | --- | --- | --- | --- | --- | --- | --- | --- |
|  |  | **Lower** | **Upper** |  |  |  | **Lower** | **Upper** |  |
| BMI | 0.335 | 0.076 | 0.594 | **0.011** | FT3 | 0.004 | -0.022 | 0.03 | 0.741 |
| Smoking  History | 0.195 | -1.176 | 1.566 | 0.78 | TSH | 0.051 | -0.01 | 0.112 | 0.102 |
| SBP | 0.02 | -0.054 | 0.093 | 0.559 | TC | 0.726 | 0.03 | 1.421 | **0.041** |
| DBP | 0.046 | -0.066 | 0.158 | 0.421 | TG | 0.832 | -0.016 | 1.68 | **0.054** |
| TPOAb | 9.53E-05 | -0.001 | 0.001 | 0.773 | HDL-c | -0.232 | -2.67 | 2.206 | 0.851 |
| TGAb | 0.001 | -0.001 | 0.003 | 0.476 | LDL-c | 0.995 | 0.17 | 1.819 | **0.018** |
| TMAb | 0.019 | -0.048 | 0.086 | 0.576 | ApoA | 1.793 | -1.878 | 5.465 | 0.337 |
| T4 | 0.126 | -0.134 | 0.385 | 0.342 | ApoB | 2.514 | -0.776 | 5.803 | 0.133 |
| T3 | -0.029 | -0.349 | 0.291 | 0.859 | ApoE | 0.048 | -0.003 | 0.099 | **0.066** |
| FT4 | -0.046 | -0.092 | -0.001 | **0.047** | Lp(a) | 0 | -0.004 | 0.004 | 0.952 |

**Abbreviations:** BMI, body mass index; SBP, systolic blood pressure; DBP, diastolic blood pressure; ATD, Antithyroxine drugs; T4, Thyroxine; T3, Triiodothyronine; TSH, Thyrotropin; TG, triglyceride; TC, total cholesterol; APOA, apolipoprotein A; APOB, apolipoprotein B; ApoE, apolipoprotein E;Lp(a), Lipoprotein(a); HDL-c, high-density lipoprotein cholesterol; LDL-c, low-density lipoprotein cholesterol;
